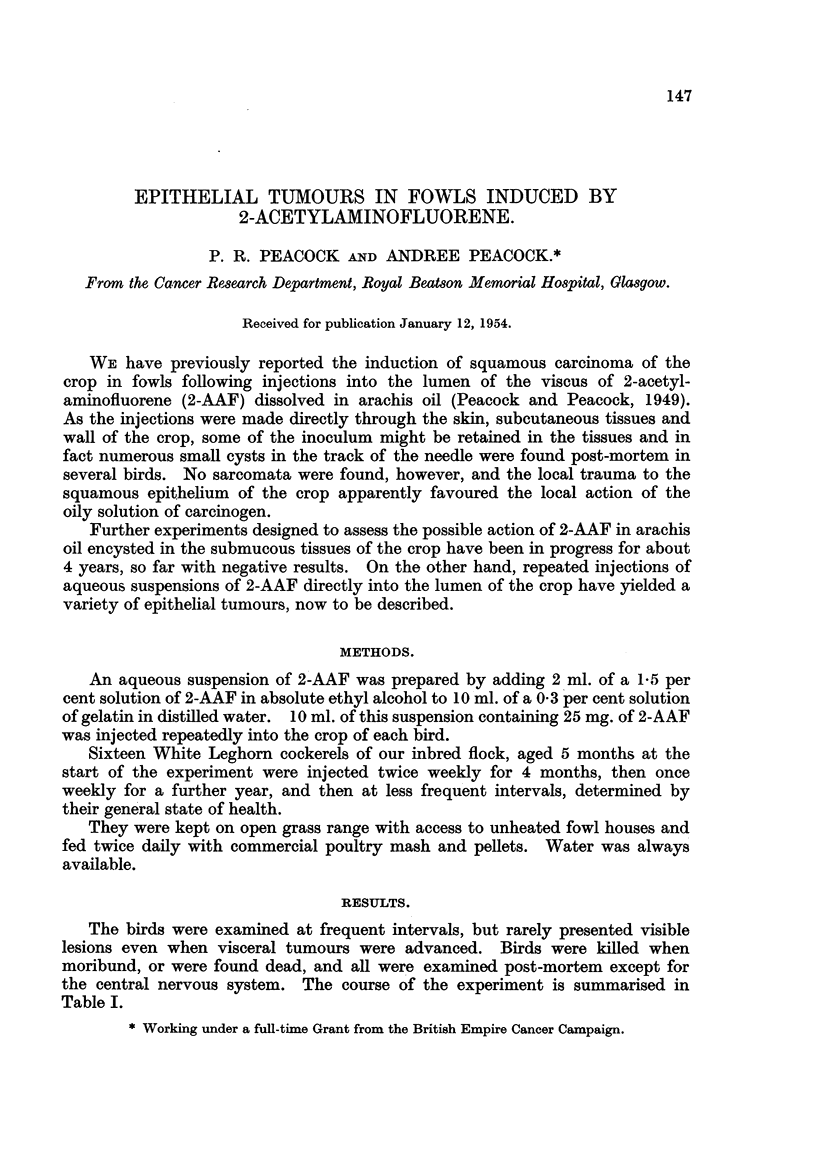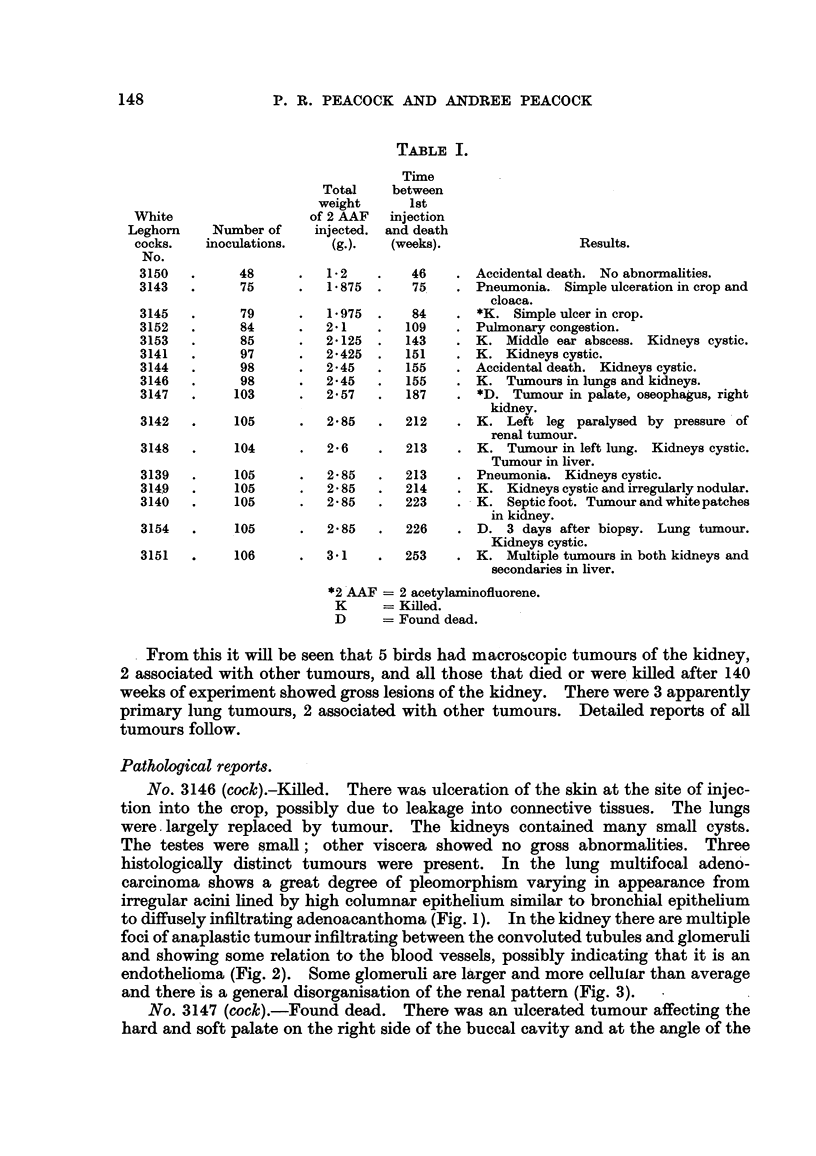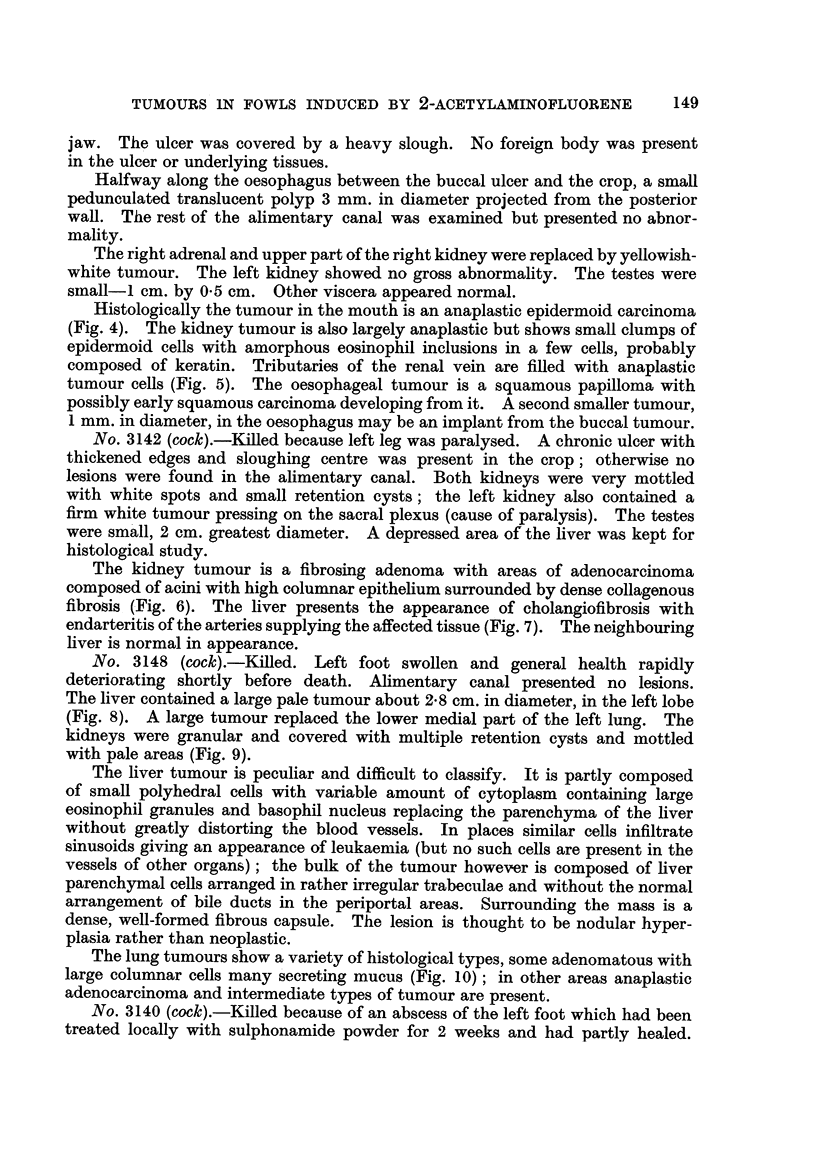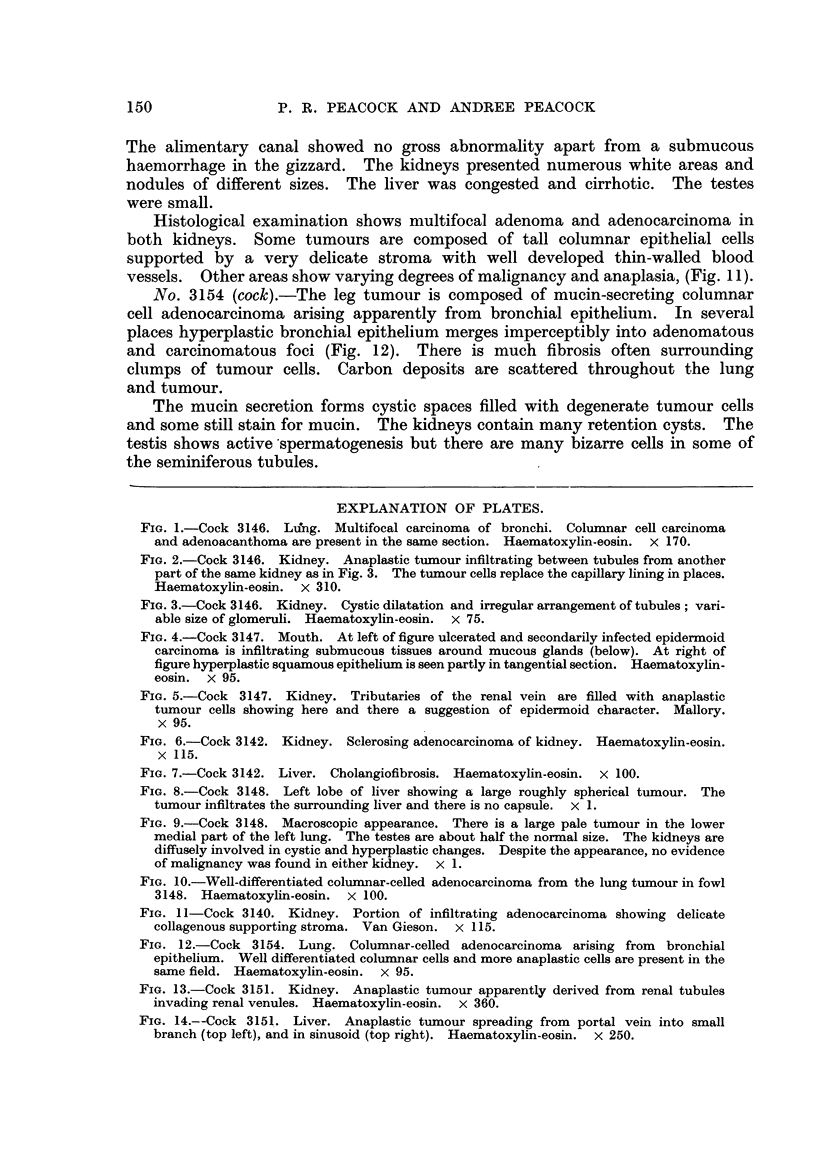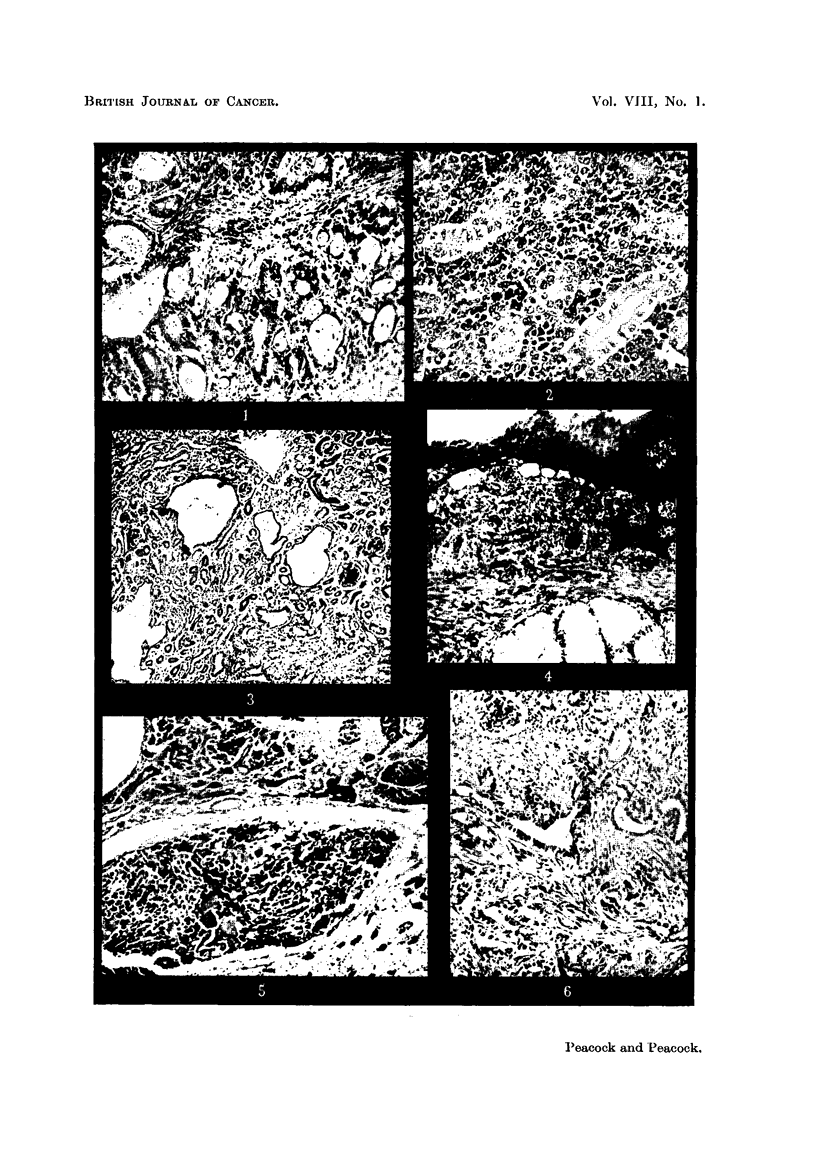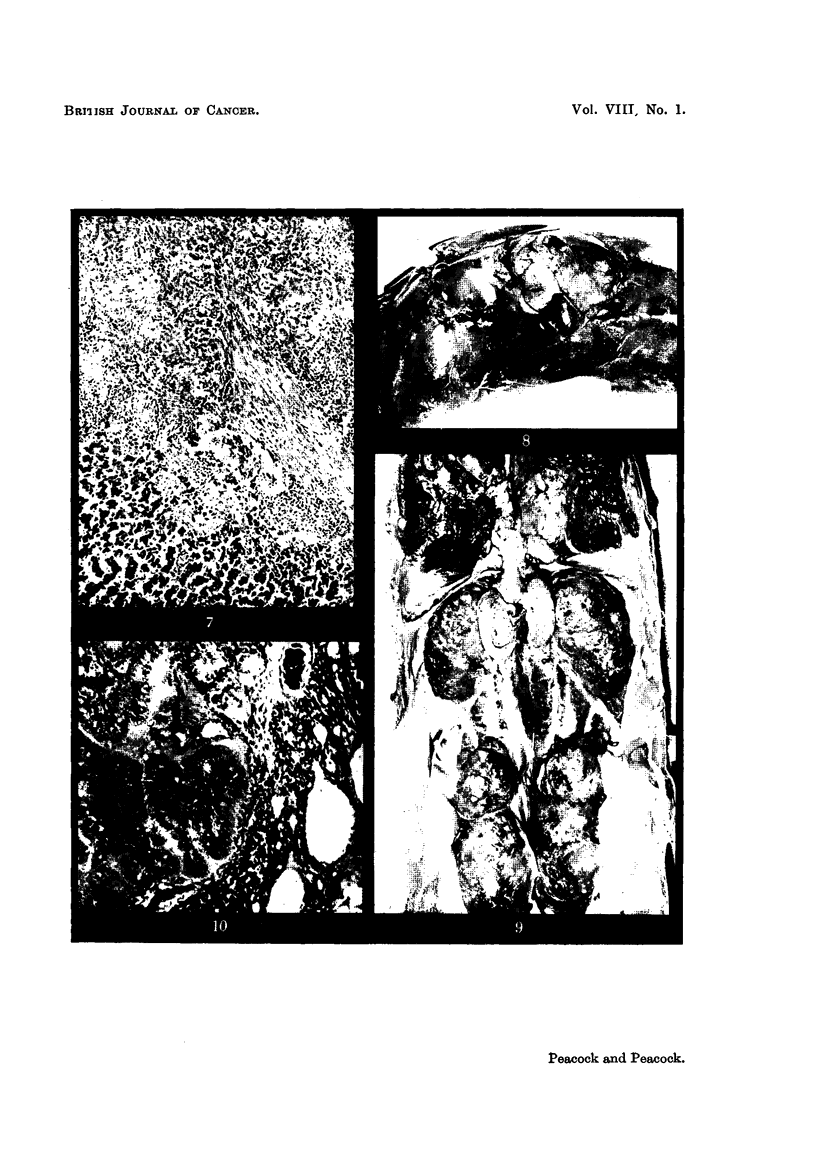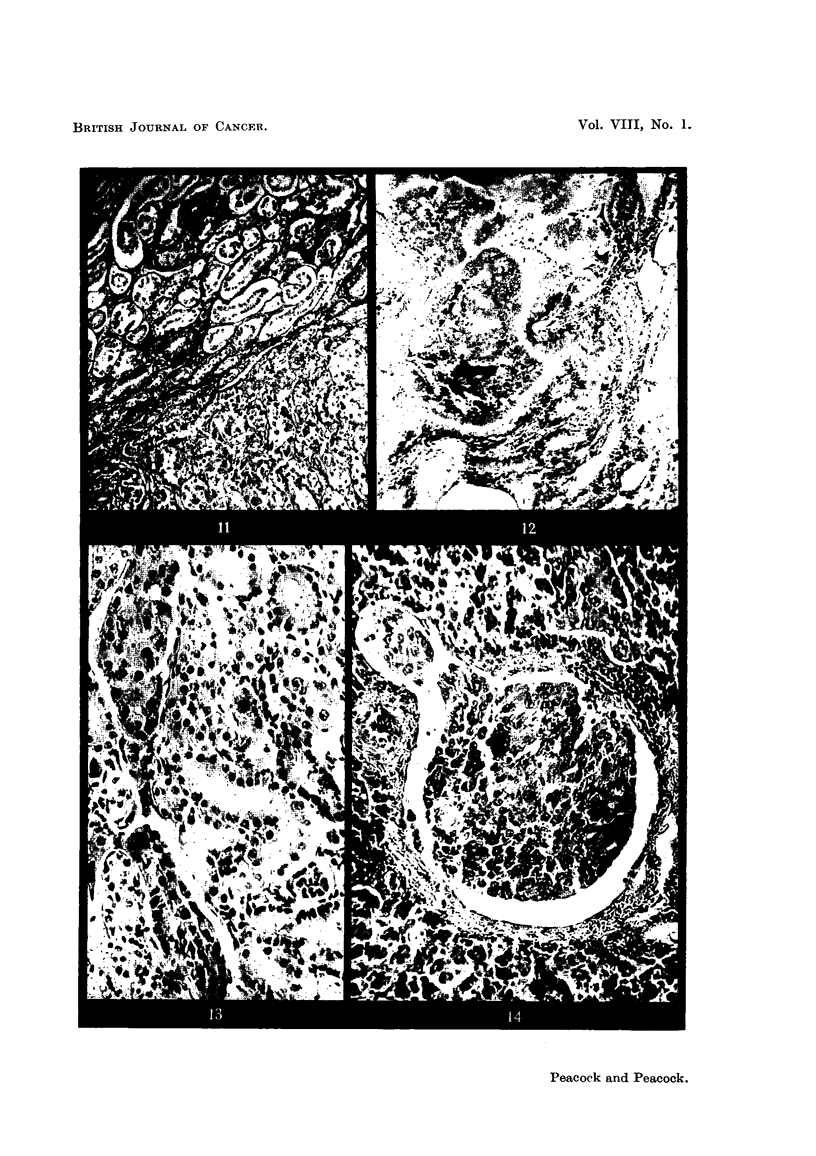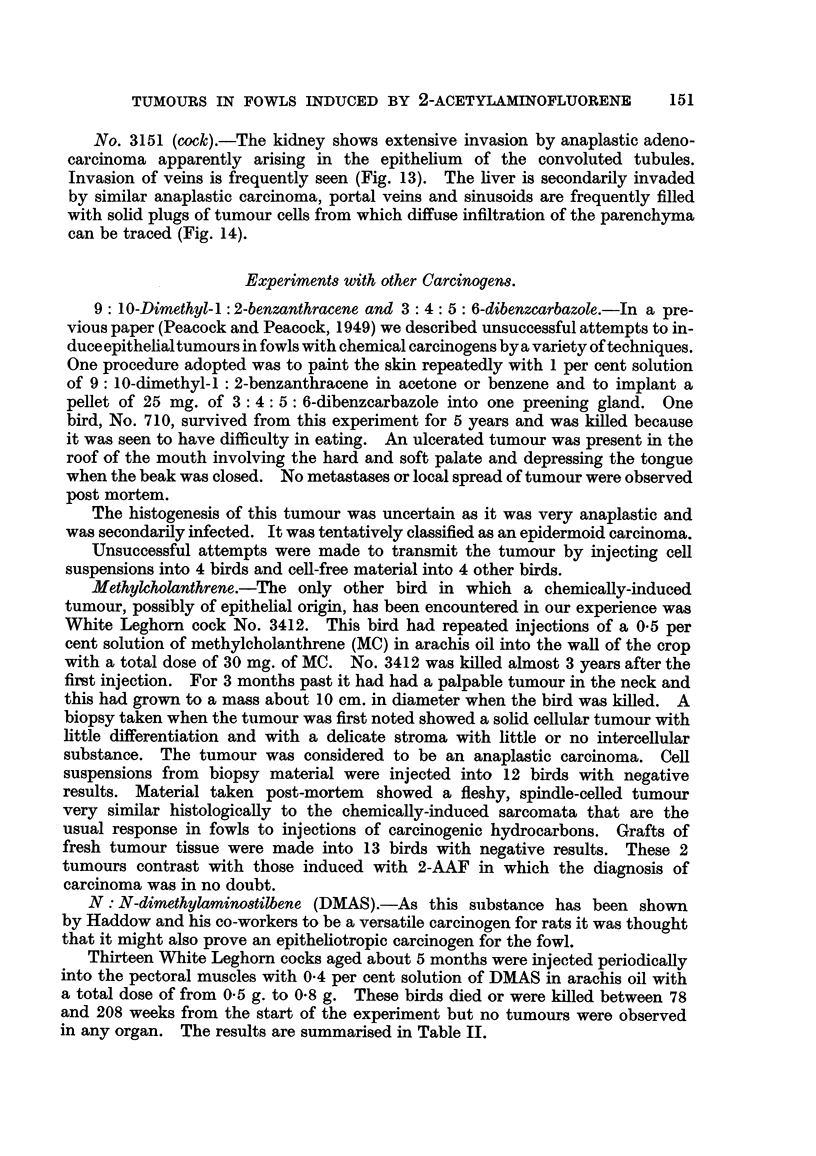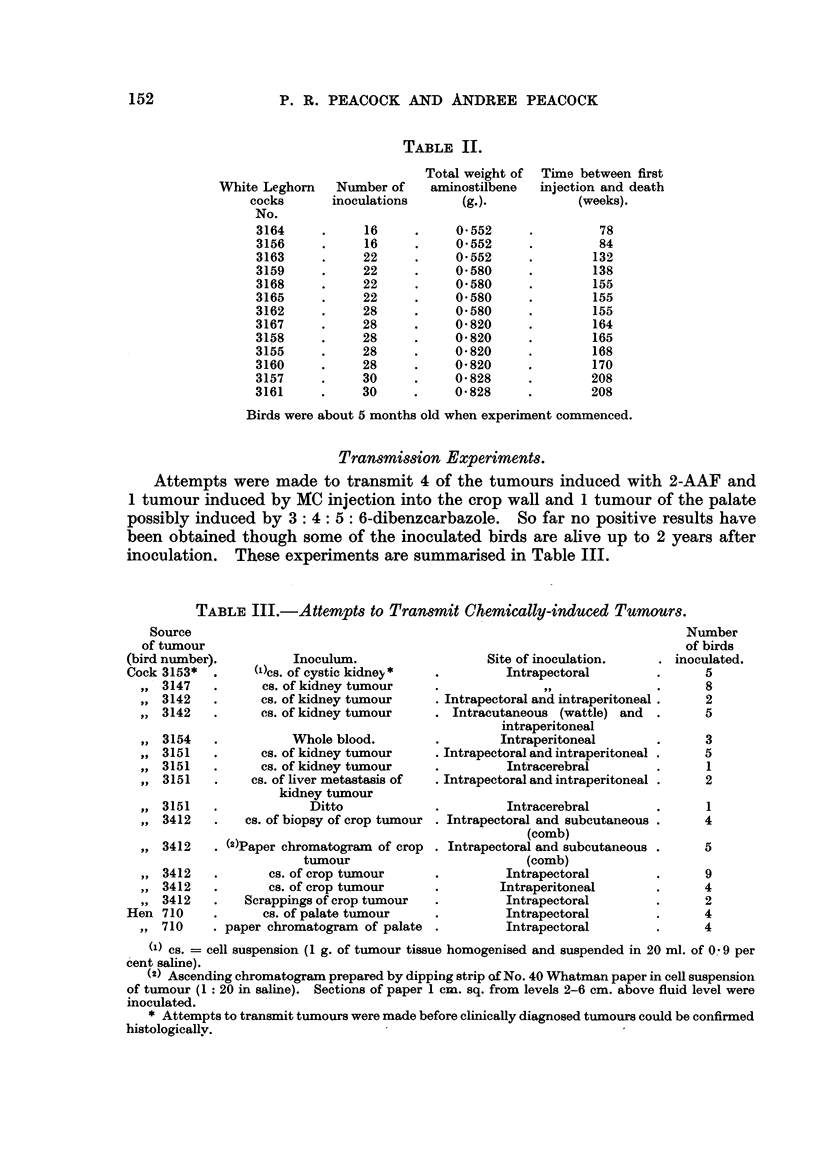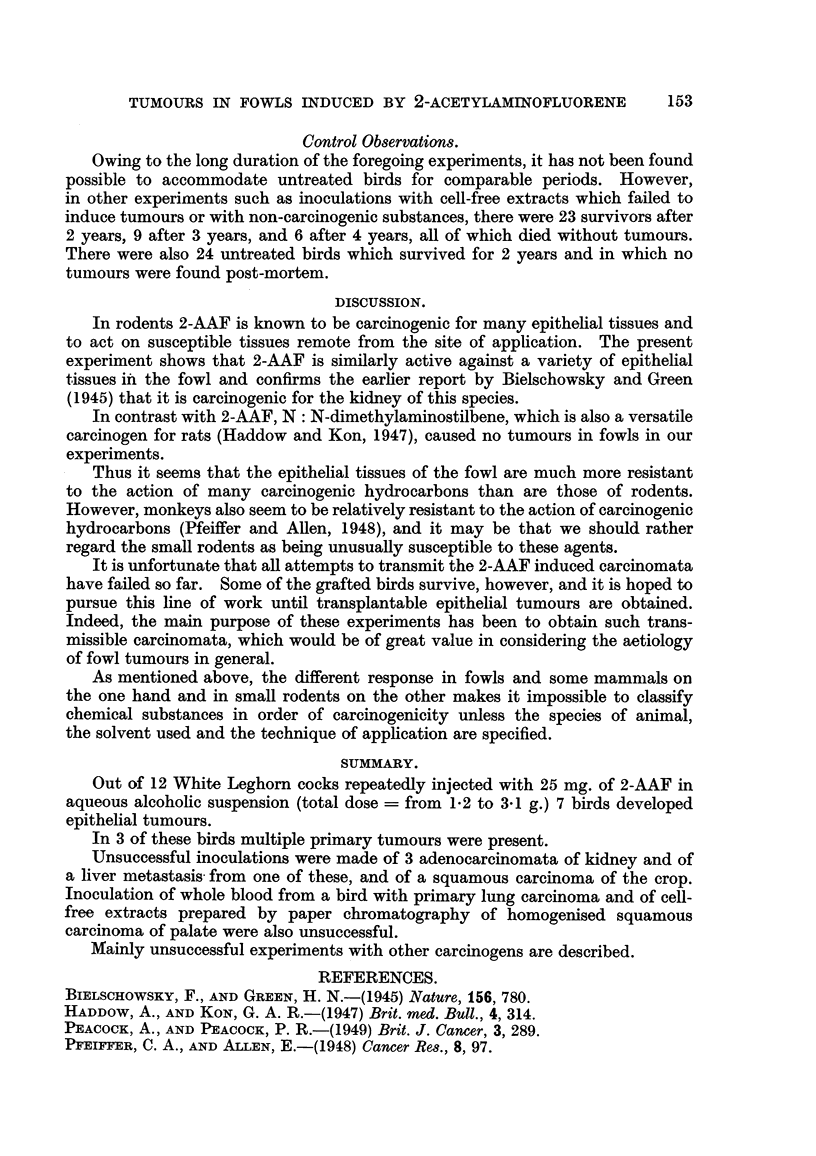# Epithelial Tumours in Fowls Induced by 2-Acetylaminofluorene

**DOI:** 10.1038/bjc.1954.12

**Published:** 1954-03

**Authors:** P. R. Peacock, Andree Peacock

## Abstract

**Images:**


### EPITHELIAL TUMOURS IN FOWLS INDUCED BY 2-ACETYLAMINOFLUORENE.

P. R. PEACOCK AND ANDREE PEACOCK.*

*From the Cancer Research Department, Royal Beatson Memorial Hospital, Glasgow.*

Received for publication January 12, 1954.

WE have previously reported the induction of squamous carcinoma of the crop in fowls following injections into the lumen of the viscus of 2-acetylaminofluorene (2-AAF) dissolved in arachis oil (Peacock and Peacock, 1949). As the injections were made directly through the skin, subcutaneous tissues and wall of the crop, some of the inoculum might be retained in the tissues and in fact numerous small cysts in the track of the needle were found post-mortem in several birds. No sarcomata were found, however, and the local trauma to the squamous epithelium of the crop apparently favoured the local action of the oily solution of carcinogen.

Further experiments designed to assess the possible action of 2-AAF in arachis oil encysted in the submucous tissues of the crop have been in progress for about 4 years, so far with negative results. On the other hand, repeated injections of aqueous suspensions of 2-AAF directly into the lumen of the crop have yielded a variety of epithelial tumours, now to be described.

### METHODS.

An aqueous suspension of 2-AAF was prepared by adding 2 ml. of a 1·5 per cent solution of 2-AAF in absolute ethyl alcohol to 10 ml. of a 0·3 per cent solution of gelatin in distilled water. 10 ml. of this suspension containing 25 mg. of 2-AAF was injected repeatedly into the crop of each bird.

Sixteen White Leghorn cockerels of our inbred flock, aged 5 months at the start of the experiment were injected twice weekly for 4 months, then once weekly for a further year, and then at less frequent intervals, determined by their general state of health.

They were kept on open grass range with access to unheated fowl houses and fed twice daily with commercial poultry mash and pellets. Water was always available.

### RESULTS.

The birds were examined at frequent intervals, but rarely presented visible lesions even when visceral tumours were advanced. Birds were killed when moribund, or were found dead, and all were examined post-mortem except for the central nervous system. The course of the experiment is summarised in Table I.

* Working under a full-time Grant from the British Empire Cancer Campaign.

TABLE I.

| White Leghorn cocks. No. | Number of inoculations. | Total weight of 2 AAF injected. (g.). | Time between 1st injection and death (weeks). | Results. |
|---|---|---|---|---|
| 3150 | 48 | 1·2 | 46 | Accidental death. No abnormalities. |
| 3143 | 75 | 1·875 | 75 | Pneumonia. Simple ulceration in crop and cloaca. |
| 3145 | 79 | 1·975 | 84 | *K. Simple ulcer in crop. |
| 3152 | 84 | 2·1 | 109 | Pulmonary congestion. |
| 3153 | 85 | 2·125 | 143 | K. Middle ear abscess. Kidneys cystic. |
| 3141 | 97 | 2·425 | 151 | K. Kidneys cystic. |
| 3144 | 98 | 2·45 | 155 | Accidental death. Kidneys cystic. |
| 3146 | 98 | 2·45 | 155 | K. Tumours in lungs and kidneys. |
| 3147 | 103 | 2·57 | 187 | *D. Tumour in palate, oseophagus, right kidney. |
| 3142 | 105 | 2·85 | 212 | K. Left leg paralysed by pressure of renal tumour. |
| 3148 | 104 | 2·6 | 213 | K. Tumour in left lung. Kidneys cystic. Tumour in liver. |
| 3139 | 105 | 2·85 | 213 | Pneumonia. Kidneys cystic. |
| 3149 | 105 | 2·85 | 214 | K. Kidneys cystic and irregularly nodular. |
| 3140 | 105 | 2·85 | 223 | K. Septic foot. Tumour and white patches in kidney. |
| 3154 | 105 | 2·85 | 226 | D. 3 days after biopsy. Lung tumour. Kidneys cystic. |
| 3151 | 106 | 3·1 | 253 | K. Multiple tumours in both kidneys and secondaries in liver. |

*2 AAF = 2 acetylaminofluorene.
K = Killed.
D = Found dead.

From this it will be seen that 5 birds had macroscopic tumours of the kidney, 2 associated with other tumours, and all those that died or were killed after 140 weeks of experiment showed gross lesions of the kidney. There were 3 apparently primary lung tumours, 2 associated with other tumours. Detailed reports of all tumours follow.

*Pathological reports.*

*No.* 3146 (*cock*).–Killed. There was ulceration of the skin at the site of injection into the crop, possibly due to leakage into connective tissues. The lungs were largely replaced by tumour. The kidneys contained many small cysts. The testes were small; other viscera showed no gross abnormalities. Three histologically distinct tumours were present. In the lung multifocal adenocarcinoma shows a great degree of pleomorphism varying in appearance from irregular acini lined by high columnar epithelium similar to bronchial epithelium to diffusely infiltrating adenoacanthoma (Fig. 1). In the kidney there are multiple foci of anaplastic tumour infiltrating between the convoluted tubules and glomeruli and showing some relation to the blood vessels, possibly indicating that it is an endothelioma (Fig. 2). Some glomeruli are larger and more cellular than average and there is a general disorganisation of the renal pattern (Fig. 3).

*No.* 3147 (*cock*).—Found dead. There was an ulcerated tumour affecting the hard and soft palate on the right side of the buccal cavity and at the angle of the jaw. The ulcer was covered by a heavy slough. No foreign body was present in the ulcer or underlying tissues.

Halfway along the oesophagus between the buccal ulcer and the crop, a small pedunculated translucent polyp 3 mm. in diameter projected from the posterior wall. The rest of the alimentary canal was examined but presented no abnormality.

The right adrenal and upper part of the right kidney were replaced by yellowish-white tumour. The left kidney showed no gross abnormality. The testes were small—1 cm. by 0·5 cm. Other viscera appeared normal.

Histologically the tumour in the mouth is an anaplastic epidermoid carcinoma (Fig. 4). The kidney tumour is also largely anaplastic but shows small clumps of epidermoid cells with amorphous eosinophil inclusions in a few cells, probably composed of keratin. Tributaries of the renal vein are filled with anaplastic tumour cells (Fig. 5). The oesophageal tumour is a squamous papilloma with possibly early squamous carcinoma developing from it. A second smaller tumour, 1 mm. in diameter, in the oesophagus may be an implant from the buccal tumour.

*No.* 3142 (*cock*).—Killed because left leg was paralysed. A chronic ulcer with thickened edges and sloughing centre was present in the crop; otherwise no lesions were found in the alimentary canal. Both kidneys were very mottled with white spots and small retention cysts; the left kidney also contained a firm white tumour pressing on the sacral plexus (cause of paralysis). The testes were small, 2 cm. greatest diameter. A depressed area of the liver was kept for histological study.

The kidney tumour is a fibrosing adenoma with areas of adenocarcinoma composed of acini with high columnar epithelium surrounded by dense collagenous fibrosis (Fig. 6). The liver presents the appearance of cholangiofibrosis with endarteritis of the arteries supplying the affected tissue (Fig. 7). The neighbouring liver is normal in appearance.

*No.* 3148 (*cock*).—Killed. Left foot swollen and general health rapidly deteriorating shortly before death. Alimentary canal presented no lesions. The liver contained a large pale tumour about 2·8 cm. in diameter, in the left lobe (Fig. 8). A large tumour replaced the lower medial part of the left lung. The kidneys were granular and covered with multiple retention cysts and mottled with pale areas (Fig. 9).

The liver tumour is peculiar and difficult to classify. It is partly composed of small polyhedral cells with variable amount of cytoplasm containing large eosinophil granules and basophil nucleus replacing the parenchyma of the liver without greatly distorting the blood vessels. In places similar cells infiltrate sinusoids giving an appearance of leukaemia (but no such cells are present in the vessels of other organs); the bulk of the tumour however is composed of liver parenchymal cells arranged in rather irregular trabeculae and without the normal arrangement of bile ducts in the periportal areas. Surrounding the mass is a dense, well-formed fibrous capsule. The lesion is thought to be nodular hyperplasia rather than neoplastic.

The lung tumours show a variety of histological types, some adenomatous with large columnar cells many secreting mucus (Fig. 10); in other areas anaplastic adenocarcinoma and intermediate types of tumour are present.

*No.* 3140 (*cock*).—Killed because of an abscess of the left foot which had been treated locally with sulphonamide powder for 2 weeks and had partly healed.

The alimentary canal showed no gross abnormality apart from a submucous haemorrhage in the gizzard. The kidneys presented numerous white areas and nodules of different sizes. The liver was congested and cirrhotic. The testes were small.

Histological examination shows multifocal adenoma and adenocarcinoma in both kidneys. Some tumours are composed of tall columnar epithelial cells supported by a very delicate stroma with well developed thin-walled blood vessels. Other areas show varying degrees of malignancy and anaplasia, (Fig. 11).

*No.* 3154 (*cock*).—The leg tumour is composed of mucin-secreting columnar cell adenocarcinoma arising apparently from bronchial epithelium. In several places hyperplastic bronchial epithelium merges imperceptibly into adenomatous and carcinomatous foci (Fig. 12). There is much fibrosis often surrounding clumps of tumour cells. Carbon deposits are scattered throughout the lung and tumour.

The mucin secretion forms cystic spaces filled with degenerate tumour cells and some still stain for mucin. The kidneys contain many retention cysts. The testis shows active spermatogenesis but there are many bizarre cells in some of the seminiferous tubules.

---

### EXPLANATION OF PLATES.

Fig. 1.—Cock 3146. Lung. Multifocal carcinoma of bronchi. Columnar cell carcinoma and adenoacanthoma are present in the same section. Haematoxylin-eosin. × 170.

Fig. 2.—Cock 3146. Kidney. Anaplastic tumour infiltrating between tubules from another part of the same kidney as in Fig. 3. The tumour cells replace the capillary lining in places. Haematoxylin-eosin. × 310.

Fig. 3.—Cock 3146. Kidney. Cystic dilatation and irregular arrangement of tubules ; variable size of glomeruli. Haematoxylin-eosin. × 75.

Fig. 4.—Cock 3147. Mouth. At left of figure ulcerated and secondarily infected epidermoid carcinoma is infiltrating submucous tissues around mucous glands (below). At right of figure hyperplastic squamous epithelium is seen partly in tangential section. Haematoxylin-eosin. × 95.

Fig. 5.—Cock 3147. Kidney. Tributaries of the renal vein are filled with anaplastic tumour cells showing here and there a suggestion of epidermoid character. Mallory. × 95.

Fig. 6.—Cock 3142. Kidney. Sclerosing adenocarcinoma of kidney. Haematoxylin-eosin. × 115.

Fig. 7.—Cock 3142. Liver. Cholangiofibrosis. Haematoxylin-eosin. × 100.

Fig. 8.—Cock 3148. Left lobe of liver showing a large roughly spherical tumour. The tumour infiltrates the surrounding liver and there is no capsule. × 1.

Fig. 9.—Cock 3148. Macroscopic appearance. There is a large pale tumour in the lower medial part of the left lung. The testes are about half the normal size. The kidneys are diffusely involved in cystic and hyperplastic changes. Despite the appearance, no evidence of malignancy was found in either kidney. × 1.

Fig. 10.—Well-differentiated columnar-celled adenocarcinoma from the lung tumour in fowl 3148. Haematoxylin-eosin. × 100.

Fig. 11—Cock 3140. Kidney. Portion of infiltrating adenocarcinoma showing delicate collagenous supporting stroma. Van Gieson. × 115.

Fig. 12.—Cock 3154. Lung. Columnar-celled adenocarcinoma arising from bronchial epithelium. Well differentiated columnar cells and more anaplastic cells are present in the same field. Haematoxylin-eosin. × 95.

Fig. 13.—Cock 3151. Kidney. Anaplastic tumour apparently derived from renal tubules invading renal venules. Haematoxylin-eosin. × 360.

Fig. 14.—Cock 3151. Liver. Anaplastic tumour spreading from portal vein into small branch (top left), and in sinusoid (top right). Haematoxylin-eosin. × 250.

Peacock and Peacock.

7

8

9

10

11

12

13

14

*No.* 3151 (*cock*).—The kidney shows extensive invasion by anaplastic adenocarcinoma apparently arising in the epithelium of the convoluted tubules. Invasion of veins is frequently seen (Fig. 13). The liver is secondarily invaded by similar anaplastic carcinoma, portal veins and sinusoids are frequently filled with solid plugs of tumour cells from which diffuse infiltration of the parenchyma can be traced (Fig. 14).

*Experiments with other Carcinogens.*

9 : 10-*Dimethyl*-1 : 2-*benzanthracene and* 3 : 4 : 5 : 6-*dibenzcarbazole.*—In a previous paper (Peacock and Peacock, 1949) we described unsuccessful attempts to induce epithelial tumours in fowls with chemical carcinogens by a variety of techniques. One procedure adopted was to paint the skin repeatedly with 1 per cent solution of 9 : 10-dimethyl-1 : 2-benzanthracene in acetone or benzene and to implant a pellet of 25 mg. of 3 : 4 : 5 : 6-dibenzcarbazole into one preening gland. One bird, No. 710, survived from this experiment for 5 years and was killed because it was seen to have difficulty in eating. An ulcerated tumour was present in the roof of the mouth involving the hard and soft palate and depressing the tongue when the beak was closed. No metastases or local spread of tumour were observed post mortem.

The histogenesis of this tumour was uncertain as it was very anaplastic and was secondarily infected. It was tentatively classified as an epidermoid carcinoma.

Unsuccessful attempts were made to transmit the tumour by injecting cell suspensions into 4 birds and cell-free material into 4 other birds.

*Methylcholanthrene.*—The only other bird in which a chemically-induced tumour, possibly of epithelial origin, has been encountered in our experience was White Leghorn cock No. 3412. This bird had repeated injections of a 0·5 per cent solution of methylcholanthrene (MC) in arachis oil into the wall of the crop with a total dose of 30 mg. of MC. No. 3412 was killed almost 3 years after the first injection. For 3 months past it had had a palpable tumour in the neck and this had grown to a mass about 10 cm. in diameter when the bird was killed. A biopsy taken when the tumour was first noted showed a solid cellular tumour with little differentiation and with a delicate stroma with little or no intercellular substance. The tumour was considered to be an anaplastic carcinoma. Cell suspensions from biopsy material were injected into 12 birds with negative results. Material taken post-mortem showed a fleshy, spindle-celled tumour very similar histologically to the chemically-induced sarcomata that are the usual response in fowls to injections of carcinogenic hydrocarbons. Grafts of fresh tumour tissue were made into 13 birds with negative results. These 2 tumours contrast with those induced with 2-AAF in which the diagnosis of carcinoma was in no doubt.

*N : N-dimethylaminostilbene* (DMAS).—As this substance has been shown by Haddow and his co-workers to be a versatile carcinogen for rats it was thought that it might also prove an epitheliotropic carcinogen for the fowl.

Thirteen White Leghorn cocks aged about 5 months were injected periodically into the pectoral muscles with 0·4 per cent solution of DMAS in arachis oil with a total dose of from 0·5 g. to 0·8 g. These birds died or were killed between 78 and 208 weeks from the start of the experiment but no tumours were observed in any organ. The results are summarised in Table II.

TABLE II.

| White Leghorn cocks No. | Number of inoculations | Total weight of aminostilbene (g.). | Time between first injection and death (weeks). |
|---|---|---|---|
| 3164 | 16 | 0·552 | 78 |
| 3156 | 16 | 0·552 | 84 |
| 3163 | 22 | 0·552 | 132 |
| 3159 | 22 | 0·580 | 138 |
| 3168 | 22 | 0·580 | 155 |
| 3165 | 22 | 0·580 | 155 |
| 3162 | 28 | 0·580 | 155 |
| 3167 | 28 | 0·820 | 164 |
| 3158 | 28 | 0·820 | 165 |
| 3155 | 28 | 0·820 | 168 |
| 3160 | 28 | 0·820 | 170 |
| 3157 | 30 | 0·828 | 208 |
| 3161 | 30 | 0·828 | 208 |

Birds were about 5 months old when experiment commenced.

*Transmission Experiments.*

Attempts were made to transmit 4 of the tumours induced with 2-AAF and 1 tumour induced by MC injection into the crop wall and 1 tumour of the palate possibly induced by 3 : 4 : 5 : 6-dibenzcarbazole. So far no positive results have been obtained though some of the inoculated birds are alive up to 2 years after inoculation. These experiments are summarised in Table III.

TABLE III.—*Attempts to Transmit Chemically-induced Tumours.*

| Source of tumour (bird number). | Inoculum. | Site of inoculation. | Number of birds inoculated. |
|---|---|---|---|
| Cock 3153* | (1)cs. of cystic kidney* | Intrapectoral | 5 |
| „ 3147 | cs. of kidney tumour | „ | 8 |
| „ 3142 | cs. of kidney tumour | Intrapectoral and intraperitoneal | 2 |
| „ 3142 | cs. of kidney tumour | Intracutaneous (wattle) and intraperitoneal | 5 |
| „ 3154 | Whole blood. | Intraperitoneal | 3 |
| „ 3151 | cs. of kidney tumour | Intrapectoral and intraperitoneal | 5 |
| „ 3151 | cs. of kidney tumour | Intracerebral | 1 |
| „ 3151 | cs. of liver metastasis of kidney tumour | Intrapectoral and intraperitoneal | 2 |
| „ 3151 | Ditto | Intracerebral | 1 |
| „ 3412 | cs. of biopsy of crop tumour | Intrapectoral and subcutaneous (comb) | 4 |
| „ 3412 | (2)Paper chromatogram of crop tumour | Intrapectoral and subcutaneous (comb) | 5 |
| „ 3412 | cs. of crop tumour | Intrapectoral | 9 |
| „ 3412 | cs. of crop tumour | Intraperitoneal | 4 |
| „ 3412 | Scrappings of crop tumour | Intrapectoral | 2 |
| Hen 710 | cs. of palate tumour | Intrapectoral | 4 |
| „ 710 | paper chromatogram of palate | Intrapectoral | 4 |

(1) cs. = cell suspension (1 g. of tumour tissue homogenised and suspended in 20 ml. of 0·9 per cent saline).

(2) Ascending chromatogram prepared by dipping strip of No. 40 Whatman paper in cell suspension of tumour (1 : 20 in saline). Sections of paper 1 cm. sq. from levels 2–6 cm. above fluid level were inoculated.

* Attempts to transmit tumours were made before clinically diagnosed tumours could be confirmed histologically.

*Control Observations.*

Owing to the long duration of the foregoing experiments, it has not been found possible to accommodate untreated birds for comparable periods. However, in other experiments such as inoculations with cell-free extracts which failed to induce tumours or with non-carcinogenic substances, there were 23 survivors after 2 years, 9 after 3 years, and 6 after 4 years, all of which died without tumours. There were also 24 untreated birds which survived for 2 years and in which no tumours were found post-mortem.

### DISCUSSION.

In rodents 2-AAF is known to be carcinogenic for many epithelial tissues and to act on susceptible tissues remote from the site of application. The present experiment shows that 2-AAF is similarly active against a variety of epithelial tissues in the fowl and confirms the earlier report by Bielschowsky and Green (1945) that it is carcinogenic for the kidney of this species.

In contrast with 2-AAF, N : N-dimethylaminostilbene, which is also a versatile carcinogen for rats (Haddow and Kon, 1947), caused no tumours in fowls in our experiments.

Thus it seems that the epithelial tissues of the fowl are much more resistant to the action of many carcinogenic hydrocarbons than are those of rodents. However, monkeys also seem to be relatively resistant to the action of carcinogenic hydrocarbons (Pfeiffer and Allen, 1948), and it may be that we should rather regard the small rodents as being unusually susceptible to these agents.

It is unfortunate that all attempts to transmit the 2-AAF induced carcinomata have failed so far. Some of the grafted birds survive, however, and it is hoped to pursue this line of work until transplantable epithelial tumours are obtained. Indeed, the main purpose of these experiments has been to obtain such transmissible carcinomata, which would be of great value in considering the aetiology of fowl tumours in general.

As mentioned above, the different response in fowls and some mammals on the one hand and in small rodents on the other makes it impossible to classify chemical substances in order of carcinogenicity unless the species of animal, the solvent used and the technique of application are specified.

### SUMMARY.

Out of 12 White Leghorn cocks repeatedly injected with 25 mg. of 2-AAF in aqueous alcoholic suspension (total dose = from 1·2 to 3·1 g.) 7 birds developed epithelial tumours.

In 3 of these birds multiple primary tumours were present.

Unsuccessful inoculations were made of 3 adenocarcinomata of kidney and of a liver metastasis from one of these, and of a squamous carcinoma of the crop. Inoculation of whole blood from a bird with primary lung carcinoma and of cell-free extracts prepared by paper chromatography of homogenised squamous carcinoma of palate were also unsuccessful.

Mainly unsuccessful experiments with other carcinogens are described.

### REFERENCES.

Bielschowsky, F., and Green, H. N.—(1945) *Nature*, **156**, 780.
Haddow, A., and Kon, G. A. R.—(1947) *Brit. med. Bull.*, **4**, 314.
Peacock, A., and Peacock, P. R.—(1949) *Brit. J. Cancer*, **3**, 289.
Pfeiffer, C. A., and Allen, E.—(1948) *Cancer Res.*, **8**, 97.